# Transarterial radioembolization for liver tumors as neoadjuvant therapy: three case reports

**DOI:** 10.31744/einstein_journal/2020RC4990

**Published:** 2020-02-19

**Authors:** Vithor de Oliveira Carvalho, Francisco Leonardo Galastri, Breno Boueri Affonso, Priscila Mina Falsarella, Leonardo Guedes Moreira Valle, Ben-Hur Ferraz-Neto, Marcelo Bruno de Rezende, Joaquim Maurício da Motta-Leal-Filho, Rodrigo Gobbo Garcia, Felipe Nasser

**Affiliations:** 1 Hospital Israelita Albert Einstein São PauloSP Brazil Hospital Israelita Albert Einstein , São Paulo , SP , Brazil .; 2 Hospital Santa Marcelina São PauloSP Brazil Hospital Santa Marcelina , São Paulo , SP , Brazil .

**Keywords:** Radioembolization, Liver neoplasms, Cholangiocarcinoma, Neoadjuvant therapy, Embolization, therapeutic/methods

## Abstract

Transarterial radioembolization (TARE) with yttrium-90 microspheres is a palliative locoregional treatment, minimally invasive for liver tumors. The neoadjuvant aim of this treatment is still controversial, however, selected cases with lesions initially considered unresectable have been enframed as candidates for curative therapy after hepatic transarterial radioembolization. We report three cases in which the hepatic transarterial radioembolization was used as neoadjuvant therapy in an effective way, allowing posterior potentially curative therapies.

## INTRODUCTION

Primary and secondary liver cancer are important cause of death. Diagnosis and curative treatment are only possible for minority of patients, and a number of palliative treatment modalities seem to be the best therapeutic proposals indicated for treatment of most of patients. ^( 1 - 3 )^

Transarterial radioembolization (TARE) of the liver with yttrium-90 microspheres constitutes a minimally invasive locoregional palliative treatment modality. In general, indication for this procedure occurs due to progression of liver lesions, in cases of failure with conventional systemic therapy, surgical therapy (resection or transplantation) or even other locoregional modalities, such as chemoembolization or ablation. ^( 1 , 4 , 5 )^ The neoadjuvant objective of this treatment is still controversy, but selected cases of injures initially considered unresectable, when undergoing TARE, are reclassified as candidates to the curative therapy. ^( 4 , 6 , 7 )^

## OBJECTIVE

To report 3 cases of liver tumors in which transarterial radioembolization of the liver was used as a neoadjuvant therapy.

## CASE REPORT

### Case 1

**Figure 1:**
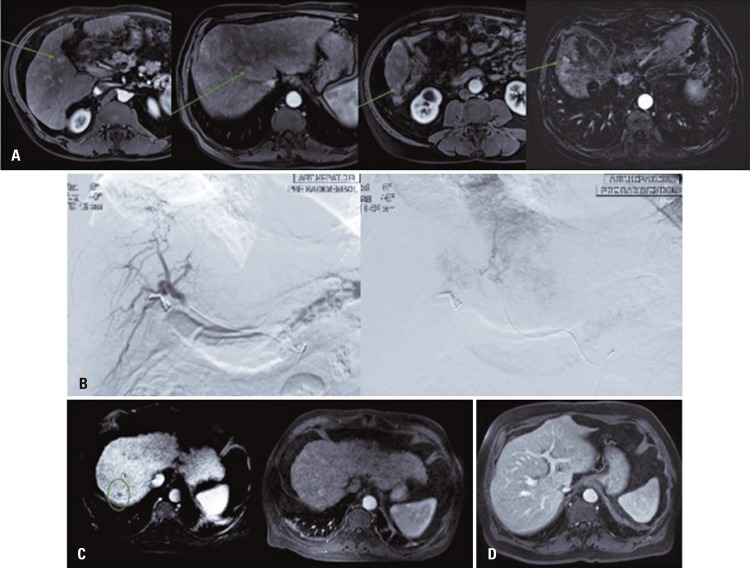
Patient with liver disease who underwent liver transplantation after transarterial radioembolization. (A) Magnetic resonance showing signs of chronic liver disease, and multiple hypervascular injuries compatible with hepatocarcinoma. Patients not within liver transplantation criteria. (B) Angiographies of liver arteries during radioembolization procedure. (C) Magnetic resonance post hepatic transarterial radioembolization: reduction and necrosis of injuries. (D) Liver transplantation on magnetic resonance

A 68-year-ol man with multinodular hepatocellular carcinoma who previously underwent TARE, radiofrequency ablation and who were not within liver transplantation criteria, and who downstaging therapy did not work ( Figure 1A ). After 7 months following the TARE ( Figure 1B ), injuries reduced in diameter with reclassification based on Milan criteria ( Figure 1C ), enabling the liver transplantation ( Figure 1D ).

### Case 2

**Figure 2:**
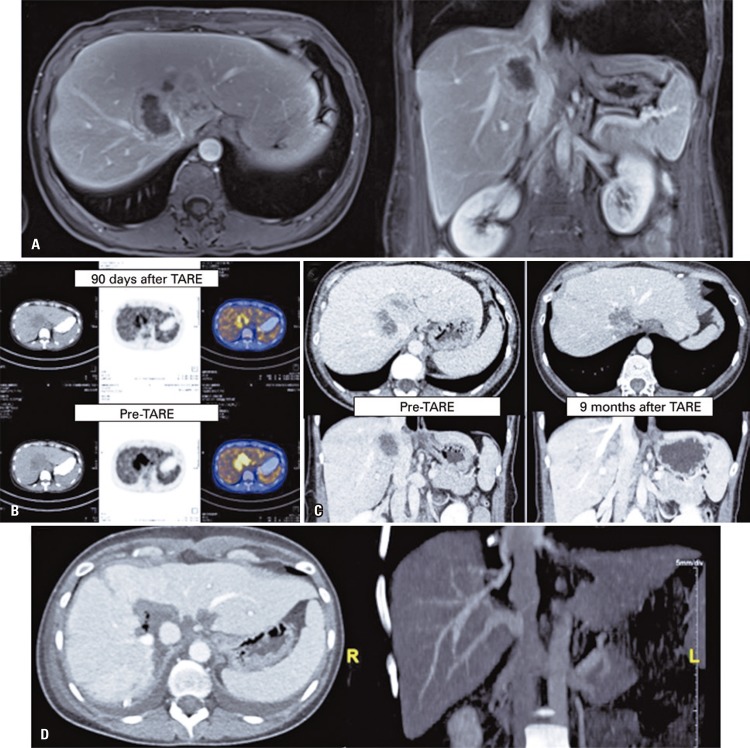
Patient with colangiocarciona who, initially, were candidate for resection. (A) Expansive injury 7.0 x 5.3cm, invading the cava vein and right and medium hepatic vein, which was suggestive of colangiomarcinoma. (B) Positron emission computed tomography conducted before and after treatment. (C) Computed tomography pre-radioembolization hepatic transarterial and follow-up (9 months after transarterial radioembolization of the liver), showing reduction of dimension of the injury and discreet contact with vascular structures. (D) Aspect of the liver in the computed tomography after resection of segments IV, VII and caudal resection

A 35-year-old woman with unresectable cholangiocarcinoma involving the right and medium liver veins, and live remanescent of 23% (lateral segment of left lobe of the liver) ( Figure 2A ). She underwent TARE and a tumor volume reduction was observed after 90 days, as well as tumor glycolytic activity (SUV=4.4; which was 10.4) ( Figure 2B ).

In the late follow-up of 9 months, we did not observe right liver vein invasion with reminiscent of 63% of the liver ( Figure 2C ), enabling the resection of affected follow-ups ( Figura 2D ).

### Case 3

**Figure 3:**
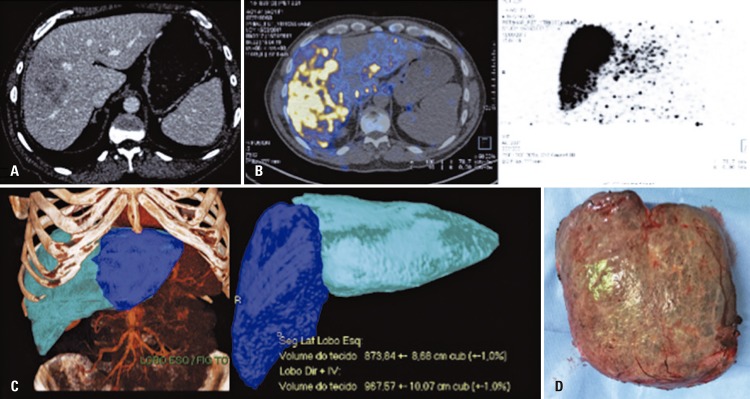
Transarterial radioembolization of the liver before surgery, showing antitumoral and ischemic effect. (A) Computed tomography with volumous suggesting hepatocarcioma on the right hepatic lobe. (B) Computed tomography by positrons emission of immediate control after transarterial radioembolization of the liver showing distribution of Y-90 in the right lobe and segment IV, and lack of uptaking in segment II and III. (C) Tomography reconstruction showing compensatory hypertrophy of left lobe after transarterial radioembolization of the liver. (D) Resected surgical specimen – right lobe and segment IV

A 59-year-old man with hepatitis C virus and voluminous hepatocellular carcinoma (7.5cm) involving segments IVa/VIII ( Figure 3A ). He was treated with TARE to control local tumor with selective radioembolization (right lobe of the liver and IV segment branch) and compensatory hypertrophy of untreated segments (II and III) ( Figure 3B ). After two months, the untreated segments represented 50% of liver volume (represented 26%) ( Figure 3C ) and patient underwent enlarged right hepatectomy ( Figure 3D ).

## DISCUSSION

In a series by Mohamed et al., comparing four different therapeutic modalities as a bridge for liver transplantation, notably TARE was effective, therefore, as the remaining, with lower advantages and lower acute toxicity (such as the stereotactic radiotherapy), enabling transplantation in patients with large in diameters injuries. ^( 8 )^

In 2011, Ettore et al., published a case report including patients in stages B and C at Barcelona Clinic Liver Cancer (BCLC) and who were not within the Milan criteria. They obtained a conversion for liver transplantation by 11%, in which we treated with TARE, initially as a palliative modality only. ^( 7 )^

In systematic review, Braat et al., presented studies that showed evidences of benefits of neodjuvant use of TARE in intra-hepatic colangiocarcinoma, in addition to hepatocarcinoma. ^( 9 )^

There were no cases of progression of the disease during time of waiting for surgical intervention. All patients reported to be with an accompanying person (mean of 25.6 months after definitive surgical treatment; ranging from 8 to 35 months) and without recurrent of the disease.

In our cases, TARE was an effective therapy, combining simultaneously the antitumor effect (cytotoxicity induced by radiation) and ischemic effect with hypertrophy of non-embolized segments, and enabling the posterior employment of potentially curative therapies. ^( 9 , 10 )^
