# Supplementary material for: Transarterial radioembolization for liver tumors as neoadjuvant therapy: three case reports
Source: Einstein (Sao Paulo). 2020 Feb 19;18:eRC4990. doi: 10.31744/einstein_journal/2020RC4990 (PMC7032884; doi:10.31744/einstein_journal/2020RC4990)
Supplement: Translation

# Translation: Radioembolização transarterial hepática como terapia neoadjuvante: relato de três casos

**Authors:** Vithor de Oliveira Carvalho, Francisco Leonardo Galastri, Breno Boueri Affonso, Priscila Mina Falsarella, Leonardo Guedes Moreira Valle, Ben-Hur Ferraz-Neto, Marcelo Bruno de Rezende, Joaquim Maurício da Motta-Leal-Filho, Rodrigo Gobbo Garcia, Felipe Nasser

**Affiliations:** 1 Brasil Hospital Israelita Albert Einstein, São Paulo, SP, Brasil .; 2 Brasil Hospital Santa Marcelina, São Paulo, SP, Brasil .

**Keywords:** Radioembolização, Neoplasias hepáticas, Colangiocarcinoma, Terapia neoadjuvante, Embolização terapêutica/métodos

## Abstract

A radioembolização transarterial hepática com microesferas de ítrio-90 é uma modalidade paliativa de tratamento locorregional minimamente invasiva. O objetivo neoadjuvante deste tratamento ainda é controverso, mas casos selecionados de lesões consideradas inicialmente irressecáveis reenquadram-se como candidatos à terapia curativa após a radioembolização transarterial hepática. Relatamos três casos em que a radioembolização transarterial hepática foi utilizada como terapia neoadjuvante de forma efetiva possibilitando aplicação posterior de terapias potencialmente curativas.

## INTRODUÇÃO

As neoplasias hepáticas primárias e secundárias são importante causa de mortalidade relacionada ao câncer. Ao diagnóstico, o tratamento curativo somente é possível em uma minoria destes pacientes, e as diversas modalidades paliativas de tratamento parecem ser as propostas terapêuticas melhores indicadas na condução da maioria destes pacientes.^( 1 - 3 )^A radioembolização transarterial hepática (TARE) com microesferas de ítrio-90 é uma modalidade paliativa de tratamento locorregional minimamente invasiva. A indicação, em geral, ocorre diante da progressão das lesões hepáticas, a despeito do uso da terapia sistêmica convencional, terapia cirúrgica (ressecção ou transplantes) ou mesmo outras modalidades locorregionais, como quimioembolização ou ablação.^( 1 , 4 , 5 )^O objetivo neoadjuvante deste tratamento ainda é controverso, mas casos selecionados de lesões consideradas inicialmente irressecáveis ao submeterem-se à TARE reenquadram-se como candidatos à terapia curativa.^( 4 , 6 , 7 )^

## OBJETIVO

Relatar três casos de tumores do fígado em que a radioembolização transarterial hepática foi utilizada como terapia neoadjuvante.

## RELATO DE CASO

### Caso 1

Homem, 68 anos, com carcinoma hepatocelular multinodular, previamente tratado com TARE e ablação por radiofrequência, fora dos critérios para transplante hepático e sem sucesso na terapia de *downstaging* ( Figura 1A ). Realizada TARE ( Figura 1B ) e, após 7 meses, as lesões demonstraram redução de diâmetro com reenquadramento aos critérios de Milão ( Figura 1C ), possibilitando o transplante hepático ( Figura 1D ).

**Figura 1:** Paciente hepatopata, submetido a transplante hepático após radioembolização transarterial hepática. (A) Ressonância magnética demonstrando sinais de hapatopatia crônica e múltiplas lesões hipervasculares compatíveis com hepatocarcinoma. Paciente fora dos critérios de transplante hepático. (B) Angiografias das artérias hepáticas durante o procedimento de radioembolização. (C) Ressonância magnética pós-radioembolização transarterial hepática: redução e necrose das lesões. (D) Ressonância magnética demonstrando fígado transplantado

### Caso 2

Paciente do sexo feminino, 35 anos, apresentando colangiocarcinoma irressecável envolvendo as veias hepáticas direita e média, e remanescente hepático de 23% (segmento lateral do lobo hepático esquerdo) ( Figura 2A ). Submetida à TARE, evidenciou redução do volume tumoral após 90 dias, bem como da atividade glicolítica tumoral (SUV=4,4; era 10,4) ( Figura 2B ). No seguimento tardio de 9 meses, não mais se evidenciava invasão da veia hepática direita, com remanescente hepático de 63% ( Figura 2C ), permitindo a ressecção dos segmentos afetados ( Figura 2D ).

**Figura 2:** Paciente portadora de colangiocarcinoma, inicialmente não candidata a ressecção. (A) Lesão expansiva de 7,0 × 5,3cm, invadindo a veia cava e veias hepáticas direita e média, sugestiva de colangiocarcinoma. (B) Tomografia computadorizada por emissão de pósitrons pré e pós-tratamento. (C) Tomografia computadorizada pré-radioembolização transarterial hepática e de seguimento (9 meses pós-radioembolização transarterial hepática), a qual demonstra redução das dimensões da lesão e discreto contato com estruturas vasculares. (D) Aspecto do fígado na tomografia computadorizada após ressecção dos segmentos IV, VIII e caudado

### Caso 3

Paciente do sexo masculino, 59 anos, hepatite vírus C e volumoso hepatocarcinoma (7,5cm) envolvendo os segmentos IVa/VIII ( Figura 3A ). Foi tratado com TARE objetivando controle tumoral local com radioembolização seletiva (lobo hepático direito e ramos do segmento IV) e hipertrofia compensatória dos segmentos não tratados (II e III) ( Figura 3B ). Dois meses após, os segmentos não tratados representavam 50% do volume hepático (representavam 26%) ( Figura 3C ) e o paciente foi submetido à hepatectomia direita ampliada ( Figura 3D ).

**Figura 3:** Radioembolização transarterial hepática pré-operatória, demonstrando efeito antitumoral e isquêmico. (A) Tomografia computadorizada com volumosa lesão sugestiva de hepatocarcinoma no lobo hepático direito. (B) Tomografia computadorizada por emissão de pósitrons de controle imediato após radioembolização transarterial hepática demonstrando distribuição do Y-90 no lobo direito e segmento IV, e ausência de captação nos segmentos II e III. (C) Reconstruções tomográficas evidenciando hipertrofia compensatória do lobo esquerdo após radioembolização transarterial hepática. (D) Peça cirúrgica ressecada − lobo direito e segmento IV

## DISCUSSÃO

Mohamed et al., em sua série comparando quatro diferentes modalidades terapêuticas como ponte para transplante hepático, notaram que a TARE foi efetiva, assim como as demais, com a vantagem de menor toxicidade aguda (tanto quanto a radioterapia estereotática), possibilitando o transplante nos pacientes com lesões de maiores diâmetros.^( 8 )^

Em 2011, Ettore et al., publicaram sua casuística de pacientes nos estágios *Barcelona Clinic Liver Cancer* (BCLC) B e C fora dos critérios de Milão, obtendo conversão para o transplante hepático em 11%, os quais foram tratados com TARE, inicialmente apenas como modalidade paliativa.^( 7 )^

Em revisão sistemática, Braat et al., apresentaram estudos que apontam evidências do benefício do uso neoadjuvante da TARE no colangiocarcinoma intra-hepático e nas metástases hepáticas, além do hepatocarcinoma.^( 9 )^

Não houve casos de progressão de doença durante o tempo de espera para intervenção cirúrgica. Todos os pacientes relatados estão em acompanhamento (média de 25,6 meses após o tratamento cirúrgico definitivo; variando de 8 a 35 meses) e sem recidiva de doença.

Em nossos casos, a TARE foi uma terapia efetiva, combinando simultaneamente o efeito antitumoral (citotoxicidade induzida pela radiação) e o efeito isquêmico com hipertrofia dos segmentos não embolizados, possibilitando o emprego posterior de terapias potencialmente curativas.^( 9 , 10 )^
